# [Corrigendum] Imatinib mesylate and nilotinib decrease synthesis of bone matrix *in vitro*

**DOI:** 10.3892/ol.2026.15688

**Published:** 2026-06-10

**Authors:** Lysann Michaela Kroschwald, Josephine Tabea Tauer, Sonja Ingrid Kroschwald, Meinolf Suttorp, Anne Wiedenfeld, Stefan Beissert, Andrea Bauer, Martina Rauner

Oncol Lett 18: 2102–2108, 2019; DOI: 10.3892/ol.2019.10518

Following the publication of the above paper, it was drawn to the Editor's attention by a concerned reader that, regarding the experiments showing the synthesis of mineralized bone matrix in the osteoblast-like cell line SaOS-2 in Fig. 1 on p. 2104, the ‘Day 10/Control’ and ‘Day 15/IAM’ data panels were apparently matching, suggesting that the data in this figure had been assembled incorrectly.

The authors have realized that the ‘Day 10/Control’ data panel was inadvertently copied across to show the results of the ‘Day 15/IAM’ experiment. The revised version of Fig. 1, now showing the correct data panel for the ‘Day 15/IAM’ experiment, is shown on the next page. The authors regret the error that were made while compiling the original figure, and are grateful to the editor of *Oncology Letters* for allowing them the opportunity to publish this Corrigendum. Note that this error did not have a significant impact on the conclusions reported in this study. All the authors agree with the publication of this corrigendum; furthermore, they apologize to the readership for any inconvenience caused.

## Figures and Tables

**Figure 1.**
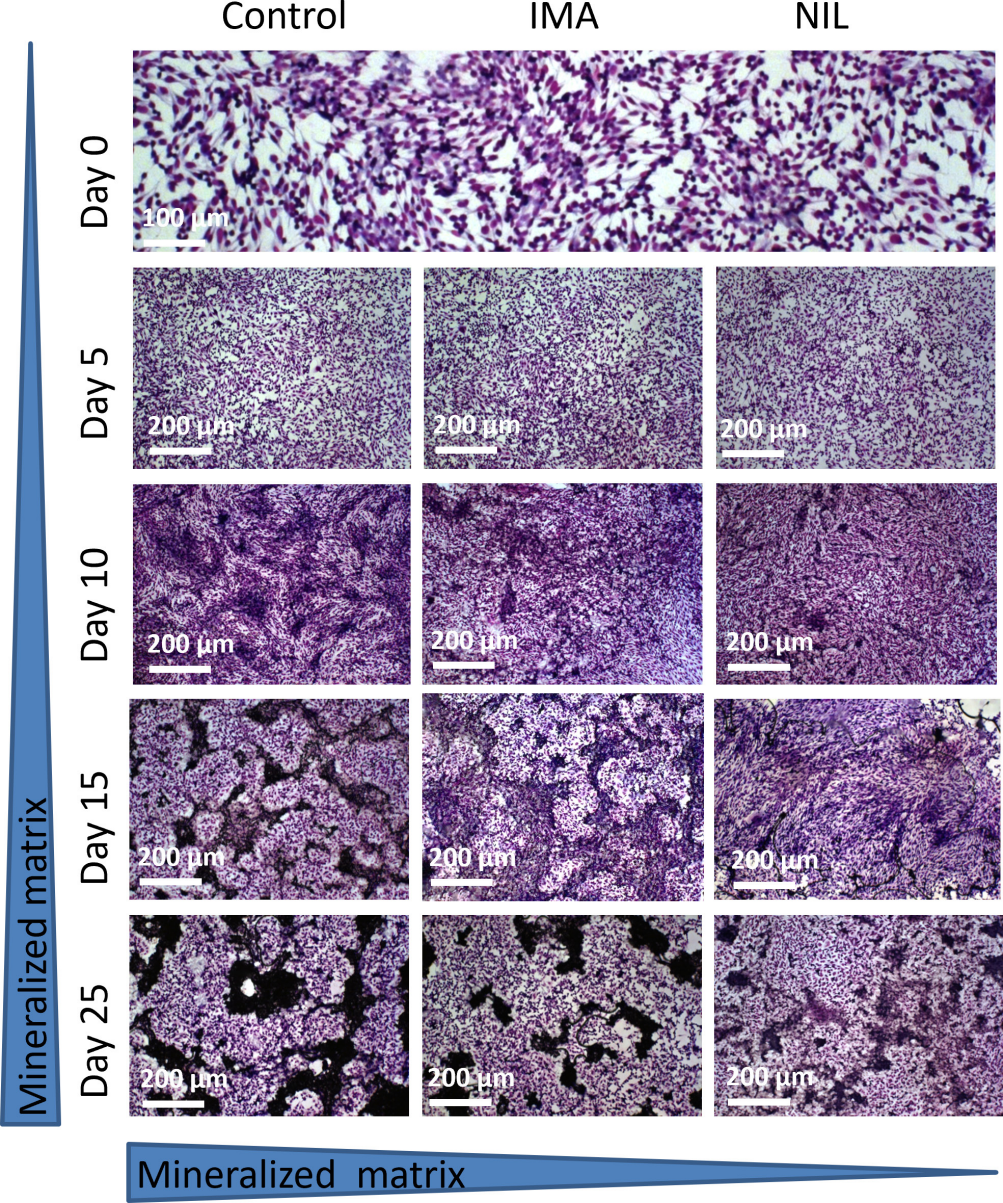
Synthesis of mineralized bone matrix in the osteoblastic-like cell line SaOS-2 under treatment with IMA (middle column, 1 µM) and NIL (1 µM, right-hand column) in comparison with untreated control (left-hand column): Hemofix-stained samples at different time points (day 0, 5, 10, 15 or 25) of incubation. Mineralized areas are represented by black areas. Representative pictures are shown (n=3). IMA, imatinib; NIL, nilotinib.

